# Analysis of Ferritin, Hepcidin, Zinc, C-Reactive Protein and IL-6 Levels in COVID-19 in Patients Living at Different Altitudes in Peru

**DOI:** 10.3390/biomedicines12071609

**Published:** 2024-07-19

**Authors:** Wilmer Silva-Caso, Sungmin Kym, Alfredo Merino-Luna, Miguel Angel Aguilar-Luis, Yordi Tarazona-Castro, Hugo Carrillo-Ng, Eliezer Bonifacio-Velez de Villa, Ronald Aquino-Ortega, Juana del Valle-Mendoza

**Affiliations:** 1 Biomedicine Laboratory, Research Center of the Faculty of Health Sciences, Universidad Peruana de Ciencias Aplicadas, Av. San Marcos Cuadra 2, Chorrillos, Lima 15023, Peru; miguel.aguilar@upc.pe (M.A.A.-L.); hugo.carrillo.ng@gmail.com (H.C.-N.); ronald.aquino@upc.pe (R.A.-O.); 2 Instituto de Investigación Nutricional, Lima 15024, Peru; 3 Division of Infectious Disease, Department of Internal Medicine, Chungnam National University School of Medicine, Daejeon 305764, Republic of Korea; 4 Unidad de Cuidados Intensivos, Clinica San Pablo, Sede Huaraz, Huaraz 02002, Peru; 5 School of Biology, Research Center of the Faculty of Health Sciences, Universidad Peruana de Ciencias Aplicdas, Lima 15023, Peru

**Keywords:** COVID-19, ferritin, hepcidin, zinc, IL-6

## Abstract

Background: Despite great scientific efforts, understanding the role of COVID-19 clinical biomarkers remains a challenge. Methods: A cross-sectional descriptive study in two Peruvian cities at different altitudes for comparison: Lima and Huaraz. In each place, three groups were formed, made up of 25 patients with COVID-19 in the ICU, 25 hospitalized patients with COVID-19 who did not require the ICU, and 25 healthy subjects as a control group. Five biomarkers were measured: IL-6, hepcidin, ferritin, C-reactive protein, and zinc using ELISA assays. Results: Ferritin, C-reactive protein, and IL-6 levels were significantly higher in the ICU and non-ICU groups at both study sites. In the case of hepcidin, the levels were significantly higher in the ICU group at both study sites compared to the non-ICU group. Among the groups within each study site, the highest altitude area presented statistically significant differences between its groups in all the markers evaluated. In the lower altitude area, differences were only observed between the groups for the zinc biomarker. Conclusion: COVID-19 patients residing at high altitudes tend to have higher levels of zinc and IL-6 in all groups studied compared to their lower altitude counterparts.

## 1. Introduction

The COVID-19 pandemic caused by the novel coronavirus SARS-CoV-2 has had a significant impact on global health since its emergence in late 2019 [1]. As of March 2023, there have been more than 758 million confirmed cases and more than 16 million deaths worldwide [2]. The virus exhibits tropism, mainly for the respiratory system, and can cause severe acute respiratory syndrome (SARS), which can lead to death in some cases [3,4]. Although most cases are mild or asymptomatic [5], there are certain risk factors that increase the likelihood of severe disease, such as age, pre-existing health conditions, and immune status [6,7].

Being able to predict the risk of developing severe disease is crucial to optimizing treatment and resource allocation [8]. Recently, more attention has been paid to proinflammatory cytokines and their role in the severity of COVID-19. There are reports of elevated serum levels of these cytokines (TNF-α, IFN-γ, IL-2, IL-4, IL-6, and IL-10) in patients with COVID-19 [9,10]. Among these, IL-6 has been described as one of those responsible for alterations in the regulation of the inflammatory process in these patients [11]. In addition, IL-6 has a role in regulating the expression of ferritin and hepcidin, proteins that regulate iron metabolism during inflammation and infections [12]. The contribution of other significant molecules in the pathogenesis of COVID-19, such as zinc, has also been investigated. This trace element is an essential micronutrient that plays a key role in immune function, in addition to having reported antiviral effects against certain pathogens, including coronaviruses [13,14].

Recent studies have described that ferritin, hepcidin, and zinc may also be involved in the pathogenesis of COVID-19, with hepcidin potentially contributing to the dysregulation of iron and zinc metabolism by playing a role in the modulation of the immune response against the virus [13,14,15]. On the other hand, high altitude has been associated with changes in the immune system and physiological adaptations that may influence the clinical outcomes of COVID-19 [16,17]. Overall, establishing the complex relationship between high altitude, IL-6, hepcidin, ferritin, C-reactive protein, and zinc in the context of COVID-19 could provide insight into the pathogenesis of the disease. Understanding this relationship is crucial to developing effective prevention and treatment strategies, particularly in high-altitude regions. Peru was severely affected by the pandemic with a high number of cases, particularly in high-altitude cities. Therefore, this research may contribute to further our understanding of the role of IL-6, ferritin, C-reactive protein, hepcidin, and zinc in COVID-19 patients residing at different altitudes.

The objective of this study was to evaluate and compare the serum levels of five biomarkers (IL-6, hepcidin, ferritin, C-reactive protein, and zinc) in patients with COVID-19 in intensive care units (ICUs), patients hospitalized with COVID-19 who did not require an ICU and healthy subjects in two Peruvian cities of different altitudes, Lima and Huaraz, to understand the relationship between altitude and COVID-19 biomarkers.

## 2. Materials and Methods

### 2.1. Study Design, Setting and Population

We carried out a descriptive cross-sectional study that aimed to analyze the levels of IL-6, hepcidin, ferritin, C-reactive protein, and zinc in groups of patients with COVID-19 from two cities located at different altitudes in Peru. In this study, all biomarkers were measured at the time of hospital admission, and the results obtained from patients in the two cities were compared. All samples and all analyses were carried out in the Biomedicine laboratory of the Universidad Peruana de Ciencias Aplicadas.

The study population consisted of patients diagnosed with COVID-19 from two cities in Peru: Huaraz and Lima during the year 2021. Huaraz is an Andean city located at an approximate altitude of 3091 m above sea level. On the other hand, Lima is an urban city, located on average at 161 m above sea level [18].

**Figure 1:**
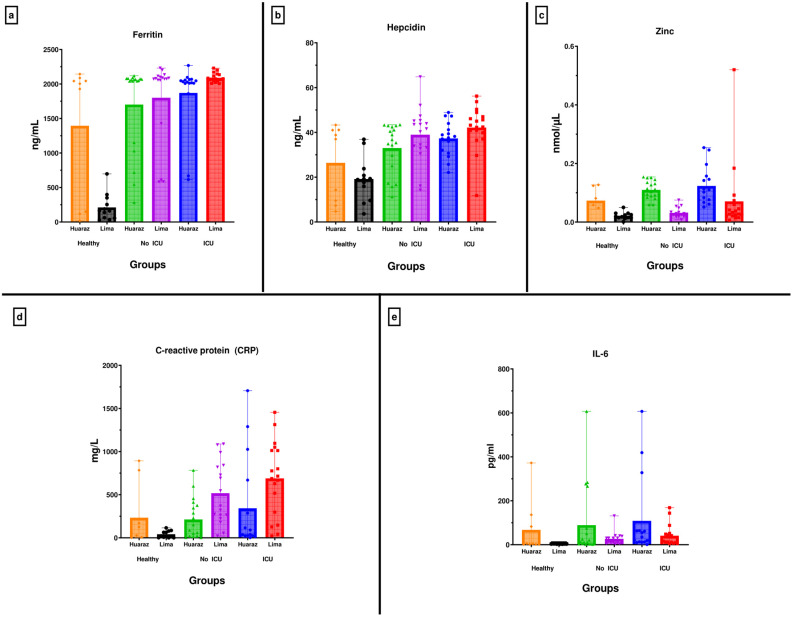
Statistic comparison of biomarkers by groups according to the altitude of the study site. (**a**) Ferritin: Mean levels, standard deviation, maximum and minimum levels, and interquartile ranges. (**b**) Hepcidin: Mean levels, standard deviation, maximum and minimum levels, and interquartile ranges. (**c**) Zinc: Mean levels, standard deviation, maximum and minimum levels, and interquartile ranges. (**d**) C-reactive protein (CPR): Mean levels, standard deviation, maximum and minimum levels, and interquartile ranges. (**e**) IL-6: Mean levels, standard deviation, maximum and minimum levels, and interquartile ranges.

Sampling was done for convenience and subjects were prospectively enrolled. The sample size was 150 individuals, taking as a reference the article by Zhou et al. [19], for a confidence level of 95% and a power of 80%. A total of 75 subjects were enrolled for each study site, the patients were recruited from a National Hospital with high-resolution capacity in Lima and a specialized Clinic in Huaraz. The study population was divided into three groups of patients for each of the cities: the ICU group [20] (25 patients with COVID-19 hospitalized in the ICU), the non-ICU group (25 patients with COVID-19 hospitalized but not eligible for the ICU), and the control group (25 healthy individuals with a negative diagnostic test for SARS-CoV-2 recruited from the community) (Figure 1). The patients enrolled in the health establishments were confirmed to have COVID-19 by a positive result of the RT-PCR test. In addition, they had a complete epidemiological record with clinical information and the declaration of residing in the place of study during the last 5 years. Patients were excluded from the study if they had a history of chronic diseases such as diabetes, hypertension, autoimmune diseases, and cardiovascular diseases to avoid confounding factors that could affect the levels of the measured biomarkers.

### 2.2. Diagnosis of COVID-19

The patients were diagnosed with COVID-19 in accordance with the protocols established by the National Institute of Health of Peru. The diagnosis was made in patients who attended with acute respiratory symptoms characterized by cough, sore throat, and at least one of the following symptoms: malaise, fever, headache, dyspnea, runny nose, or patients with respiratory distress requiring hospitalization [21].

A sample was obtained by nasopharyngeal swab from each participant and transported under standardized conditions for transporting biological material to the Laboratorio de Biomedicina de la UPC in Lima, Peru.

RNA extraction was performed using the High Pure RNA Isolation Kit (Roche Applied Science, Mannheim, Germany), following the manufacturer’s instructions. One-step RT-PCR was then performed using TaqMan with 125 nM inhibitor BHQ probe and 250 nM primers in a final volume of 20 µL. Five microliters of extracted RNA were combined with 15 μL of the master mix, and reverse transcription was performed as follows: 95 °C for 15 min, followed by 60 cycles of 15 s at 95 °C and 45 s at 60 °C. All procedures were performed on a Zcobas 480 instrument, and data were analyzed with 4.1 software (Roche Diagnostics, Mannheim, Germany). The primers and probe used were purchased from Roche Diagnostics.

### 2.3. Serum Sample Collection

Blood samples were collected from study participants after obtaining written informed consent. All samples were collected using the Vacuette TUBE Serum Separator Clot Activator (Vacuette; Greiner Bio-One, Kremsmünster, Austria). Serum samples were obtained from whole blood by centrifugation at room temperature for 20 min at 2000 rpm. Samples were then transferred to cryovials and stored at −80 °C until further processing.

### 2.4. Biochemical Analysis

IL-6, ferritin, hepcidin, C-reactive protein, and zinc levels in serum samples were assayed using commercial enzyme-linked immunosorbent assay (ELISA) kits and processed according to the manufacturer’s instructions; each assay was tested in duplicate. For the detection of IL-6, the High Sensitivity Human IL-6 ELISA kit (Abcam, Waltham, Boston, MA, USA) was used. Serum hepcidin and ferritin determination was performed using the human hepcidin and ferritin ELISA kit (Cusabio Biotech, Houston, TX, USA). Zinc and C-reactive protein levels were assessed using the Zinc Assay ELISA kit (ab102507) (Abcam, Waltham, Boston, MA, USA).

### 2.5. Statistic Analysis

Data obtained for hepcidin, C-reactive protein, ferritin, zinc, and IL-6 levels were recorded on each participant’s data collection form and compiled into an Excel v 18.0 spreadsheet for further processing and analysis. Data were analyzed using STATA v15.00 software and GraphPad Prism v8.0 software. Descriptive statistics included frequency distribution (absolute and relative) for categorical variables and measures of central tendency and dispersion for quantitative variables. For inferential statistics, a significance level of 5% was used. Two categorical variables were compared using Fisher’s exact or Chi-square test, with Fisher’s exact test being used if more than 20% of the expected values were less than 5. Student’s *t*-test or Mann–Whitney U-test was used to compare categorical variables and numerical variables, according to whether the data followed a normal distribution, as assessed by the Shapiro-Wilk and Levene tests of homogeneity of variances. If the data did not follow a normal distribution, the Mann–Whitney U-test was used.

### 2.6. Ethical Considerations

The Research Ethics Council of the Universidad Peruana de Ciencias Aplicadas of Lima, Peru (N°/FSC-CEI/646-07-21) approved this study protocol. All procedures were carried out in accordance with applicable ethical standards and guidelines. Written informed consent was obtained according to CIOMS-WHO guidelines from all participants, for the detection of SARS-CoV-2 in nasopharyngeal samples and biochemical markers in blood samples.

## 3. Results

Seventy-five participants were studied at each of the two study sites. In turn, three groups were formed in each place, consisting of 25 patients with COVID-19 in the ICU, 25 hospitalized patients with COVID-19 who did not require an ICU, and 25 healthy subjects as a control group. Mean levels, standard deviation, maximum and minimum levels, and interquartile ranges were calculated for each biomarker: ferritin, hepcidin, zinc, IL-6, and C-reactive protein (CRP) for each study group and are presented in Table 1 and Figure 1. Ferritin levels were significantly higher in the ICU groups compared to the non-ICU and control groups in both study locations, with mean levels of 1869.1 ± 485.7 ng/mL for Huaraz (3091 masl) and 2090, 1 ± 64.0 ng/mL for Lima (161 masl).

Hepcidin levels were significantly higher in the ICU groups at both study sites compared to the non-ICU and control groups, with mean values of 37.2 ± 7.8 pg/mL for Huaraz and 42.1 ± 9.8 pg/mL for Lima.

On the other hand, the levels of C-reactive protein and IL-6 were higher in the ICU groups compared to the other two groups in both study locations. Mean C-reactive protein levels were 339.3 ± 538 mg/L for Huaraz and 686.9 ± 431.2 mg/L for Lima, while mean IL-6 levels were 108.6 ± 178.9 pg/mL for Huaraz and 40.4 ± 47.7 pg/mL for Lima.

It is observed that the control group from the higher altitude place has significantly higher ferritin levels compared to the control group from the lower altitude place (1392.8 ± 975.7 ng/mL vs. 210.8 ± 194.2 ng/mL, respectively). The control group at the higher altitude site had higher hepcidin levels compared to the control group at the lower altitude site (26.3 ± 16.8 vs. 19.1 ± 9.8, respectively)

In the case of zinc, the ICU group at the higher altitude site had mean levels of 0.123 ± 0.063 nmol/µL compared to the ICU group at the lower altitude site, which showed lower zinc levels with an average of 0.070 ± 0.120 nmol/µL. Similarly, the control group in the higher altitude area presented higher zinc levels compared to the other control group, which registered average values of 0.073 ± 0.032 vs. 0.021 ± 0.012 nmol/µL.

Regarding C-reactive protein and IL-6, when both control groups were compared, we found that the control group from the higher altitude site had higher mean levels of inflammatory markers (231.5 ± 349.6 mg/L for C-reactive protein and 67.3 ± 124.0 pg/mL for IL-6), compared with the control group from the lower altitude site (39.0 ± 40.1 mg/L for CRP and 3.7 ± 0.8 pg/mL for IL-6) (Table 1).

Statistical comparison of biomarkers between groups within each study site was also performed. In the groups belonging to the highest altitude, statistically significant differences were found between the control versus the non-ICU group and the control versus the ICU group for the biomarkers ferritin, hepcidin, C-reactive protein, and IL-6. Only in the case of zinc, a difference was found only between the control group and the ICU group. At the lower altitude study site, differences were found between the control group versus the non-ICU group and the control group versus the ICU group for the zinc biomarker (Table 2).

**Figure 2:**
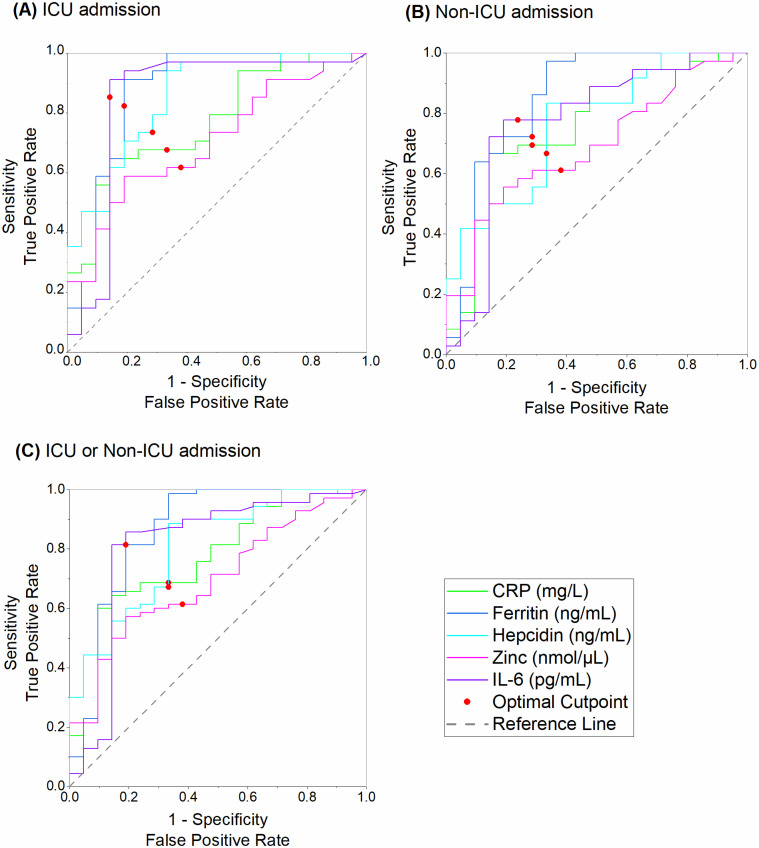
ROC curve analysis of different predictors of hospital admission in the intensive care unit (ICU) and no-ICU. ROC (receiver operating characteristic).

ROC curve analysis was performed for the different biomarkers related to intensive care unit (ICU) admission and non-ICU admission (Figure 2).

For CRP in the ICU group, an AUC of 0.76891 was found with a 95% CI of 0.639–0.898, indicating good performance with a cut-off value of 71.423 mg/L. Ferritin was found to have the highest AUC (0.87535 for ICU, with a 95% CI of 0.783–0.968 and a cut-off value of 2009.820 ng/mL), suggesting it is the best biomarker to predict admission to the ICU. On the other hand, IL-6 shows high sensitivity and specificity values for ICU admissions (85.29% and 85.71%, respectively). Hepcidin also showed good performance, with an AUC greater than 0.80, a 95% CI of 0.752–0.951, and a cut-off value of 36.715 ng/mL.

## 4. Discussion

The present study investigated the levels of different biomarkers in ICU COVID-19 patients, hospitalized COVID-19 patients who were not ICU tributaries, and healthy subjects in two different geographic regions in Peru: Lima, located at sea level, and Huaraz, located at an altitude of 3091 m above sea level. Our findings revealed that COVID-19 patients in the ICU had significantly higher levels of ferritin, hepcidin, zinc, CRP, and IL-6 compared with the other two study groups in both geographic regions.

We report that the control group living at the higher altitude had higher levels of hepcidin and ferritin compared to those living at the lower altitude. Ferritin and hepcidin are important molecules in iron metabolism. Ferritin is commonly recognized as an indicator of iron stores; however, it is also an acute phase reactant, which is regulated by proinflammatory cytokines [22]. Likewise, serum hepcidin is elevated by inflammatory cytokines, particularly IL-6 [23]. Measurement of serum hepcidin has potential as a valuable technique to assess iron levels, especially in situations where absolute and functional iron deficiency may occur concurrently [24]. In addition, hepcidin has been established as an independent prognostic indicator of mortality among patients in intensive care units, highlighting its value as an inflammatory biomarker [25]. Our findings are consistent with previous research, showing that alterations in the molecules involved in iron metabolism during COVID-19 infection lead to an unregulated inflammatory process [26]. Elevated levels of hepcidin and ferritin have been consistently reported in patients with COVID-19, particularly those with severe disease [27,28].

However, the relationship between these biomarkers, altitude, and the immune response to COVID-19 has not been fully elucidated. Previous studies have shown that iron metabolism can adapt to chronic hypoxic exposure, which is commonly seen in people living at high altitudes [29]. Some changes include increased production of erythropoietin and red blood cells, and regulation of ferritin and hepcidin levels [29,30,31,32].

Zinc is an essential micronutrient that plays a crucial role in immune function and has been identified as a potential treatment option for COVID-19 [31]. Our findings indicate that patients with severe disease (ICU group) had higher zinc levels, particularly at the higher altitude site. In addition, the control group and the non-ICU group from Huaraz had higher zinc levels compared to their Lima counterparts. This differs from previous evidence indicating a lower level of zinc in patients in severe condition [33,34]. Our findings could suggest a probable alteration of zinc metabolism at higher altitudes in the framework of the complex relationship between zinc and the severity of COVID-19. Several factors can influence serum zinc levels, including diet, fasting state, altitude, diurnal variation, exercise, and gender [34]. In fact, zinc levels have been associated with excessive erythrocytosis in men living at high altitudes, which could lead to variations in ferritin and hepcidin levels, as mentioned above [35]. Uncovering the interaction of these biomarkers may provide a better understanding of the pathogenesis of COVID-19 at higher altitudes.

Finally, we found that both IL-6 and CRP were higher in the ICU groups, particularly in patients living in Huaraz. In addition, the control group subjects living at the highest altitude had higher levels of inflammatory biomarkers compared to those living at the lowest altitude. These findings are consistent with our previous study, which showed a greater inflammatory response among patients living at higher altitudes [17]. Previous studies have shown that residents of high-altitude cities experience elevated inflammatory markers due to chronic hypoxia, even in the absence of disease [36]. This is particularly true for circulating levels of IL-6, IL-1, and CRP, which increase in response to hypobaric and hypoxic conditions experienced at higher altitudes [37]. Furthermore, it has been observed that regular exposure to high altitudes increases serum levels of acute phase proteins since in this context, inflammatory signaling pathways and immune function must respond and adapt to acute or chronic hypoxemia. There are studies with residents exposed to an altitude similar to that of our study, where the responses to inflammatory stimuli were also greater with respect to the population living at sea level. However, the high values obtained in our control group must be interpreted in light of future studies that may contemplate follow-up of individuals [36,38,39,40]. The clinical implications of these findings have yet to be elucidated.

Overall, our findings highlight the complex relationship between COVID-19, altitude, and the immune response, which also influences iron and zinc metabolism in the human body. Elucidating the interaction between these biomarkers could lead to a better understanding of the pathogenesis of COVID-19 and better clinical outcomes, particularly in patients living at different altitudes.

Limitations: Studies with small sample sizes may not be able to detect subtle differences in biomarker levels between groups, and may also limit the generalizability of the findings to larger populations. Patients were not followed up to establish the dynamics of biomarker behavior in the context of COVID-19 disease. Another limitation of the study is that demographic and clinical factors were not considered, with which a multivariate analysis could be carried out.

## 5. Conclusions

We report that the levels of ferritin, hepcidin, zinc, CRP, and IL-6 are higher in patients with COVID-19 in the ICU in the intergroup comparison in both the higher altitude region and the lower altitude region. In the comparison by altitude, patients who live at higher altitudes have higher levels of zinc and IL-6 in all groups studied compared to their counterparts at lower altitudes. Higher levels of the biomarkers hepcidin, zinc, C-reactive protein, and IL-6 were recorded in the control group from the higher altitude region compared to the control group from the lower altitude region. In the group of patients with COVID-19 in the ICU, the biomarkers ferritin, hepcidin, and C-reactive protein have higher values in the lower altitude region. The results of ferritin levels suggest that it is the best biomarker to predict ICU admission. Overall, the study highlights the importance of considering altitude and its influence on biomarker levels in COVID-19 research and clinical management.

## Figures and Tables

**Table 1:** Laboratory parameters by groups according to the altitude of the study site.

Laboratory Parameters	Statistics	Huaraz (3091 masl)				Lima (161 masl)			
		Control	No ICU	ICU	*p*-Value	Control	No ICU	ICU	*p*-Value
Ferritin (ng/mL)	Mean ± SD	1392.8 ± 975.7	1699.4 ± 615.0	1869.1 ± 485.7	0.0001	210.8 ± 194.2	1798.0 ± 600.8	2090.1 ± 64.0	0.2370
	Max	2143.9	2119.7	2269.4		698.0	2231.3	2230.5	
	Min	16.5	284.2	614.3		28.0	580.3	2006.0	
	IQR	1894.0	923.1	51.9		242.1	45.9	91.0	
Hepcidin (ng/mL)	Mean ± SD	26.3 ± 16.8	32.9 ± 10.6	37.2 ± 7.8	<0.001	19.1 ± 9.8	39.0 ± 12.2	42.1 ± 9.8	0.3221
	Max	43.3	43.4	48.9		36.9	64.8	56.2	
	Min	4.7	11.2	22.1		3.6	14.2	11.8	
	IQR	31.5	17.4	11.3		9.5	11.8	7.2	
Zinc (nmol/µL)	Mean ± SD	0.073 ± 0.032	0.109 ± 0.030	0.123 ± 0.063	0.0757	0.021 ± 0.012	0.031 ± 0.018	0.070 ± 0.120	0.0178
	Max	0.127	0.154	0.254		0.050	0.075	0.520	
	Min	0.044	0.059	0.051		0.001	0.008	0.006	
	IQR	0.028	0.046	0.076		0.013	0.015	0.039	
CRP (mg/L)	Mean ± SD	231.5 ± 349.6	209.9 ± 230.0	339.3 ± 538.3	0.0001	39.0 ± 40.1	514.7 ± 357.5	686.9 ± 431.2	0.9433
	Max	892.7	783.2	1706.3		115.6	1088.3	1455.0	
	Min	2.1	1.7	2.5		1.0	21.1	23.0	
	IQR	140.9	356.1	453.3		66.6	557.7	715.5	
IL-6 (pg/mL)	Mean ± SD	67.3 ± 124.0	88.9 ± 159.2	108.6 ± 178.9	0.0001	3.7 ± 0.8	26.4 ± 30.2	40.4 ± 47.7	0.8232
	Max	372.1	606.8	606.8		4.9	131.3	168.5	
	Min	1.2	2.4	1.2		2.7	3.3	4.7	
	IQR	81.2	72.8	50.8		1.5	22.1	38.5	

CRP-C: C-reactive protein; IL-6: interleukin 6; IQR: interquartile range. ANOVA or Kruskal–Wallis tests were used to compare groups (*p*-value < 0.05).

**Table 2:** Statistic comparison of biomarkers between each group corresponding to a study site.

*p*-Value	Statistics	Huaraz (3091 masl)			Lima (161 masl)		
		Control	No ICU	ICU	Control	No ICU	ICU
Ferritin (ng/mL)	Control	-	0.0001	0.0001	-	0.777	0.275
	No ICU	0.0001	-	0.7917	0.777	-	1.000
	ICU	0.0001	0.7917	-	0.275	1.000	-
Hepcidin (ng/mL)	Control	-	<0.001	<0.001	-	0.3628	0.1929
	No ICU	<0.001	-	1.000	0.3628	-	0.3205
	ICU	<0.001	1.000	-	0.1929	0.3205	-
Zinc (nmol/µL)	Control	-	0.1209	0.0292	-	0.0054	0.0203
	No ICU	0.1209	-	0.3727	0.0054	-	0.9736
	ICU	0.0292	0.3727	-	0.0203	0.9736	-
CRP (mg/L)	Control	-	0.0001	0.0001	-	0.7122	0.8651
	No ICU	0.0001	-	0.3221	0.7122	-	0.8946
	ICU	0.0001	0.3221	-	0.8651	0.8946	-
IL-6 (pg/mL)	Control	-	0.0001	0.0001	-	1.000	1.000
	No ICU	0.0001	-	0.2834	1.000	-	1.000
	ICU	0.0001	0.2834	-	1.000	1.000	-

ANOVA/Bonferroni or Kruskal–Wallis test between groups (*p*-value < 0.05).

## Data Availability

The datasets generated and/or analyzed during the current study are available in the figshare repository, LINK: https://doi.org/10.6084/m9.figshare.22911887.
